# Functional Gene Identification and Corresponding Tolerant Mechanism of High Furfural-Tolerant *Zymomonas mobilis* Strain F211

**DOI:** 10.3389/fmicb.2021.736583

**Published:** 2021-11-11

**Authors:** Dongsheng Hu, Zhiquan Wang, Mingxiong He, Yuanyuan Ma

**Affiliations:** ^1^Department of Biochemical Engineering, School of Chemical Engineering and Technology, Tianjin University, Tianjin, China; ^2^School of Marine Science and Technology, Tianjin University, Tianjin, China; ^3^Biomass Energy Technology Research Centre, Key Laboratory of Development and Application of Rural Renewable Energy (Ministry of Agriculture and Rural Affairs), Biogas Institute of Ministry of Agriculture and Rural Affairs, Chengdu, China

**Keywords:** *Zymomonas mobilis*, furfural, tolerance, RNA-Seq, functional investigation

## Abstract

Furfural is a major inhibitor in lignocellulose hydrolysate for *Zymomonas mobilis*. A mutant F211 strain with high furfural tolerance was obtained from our previous study. Thus, its key tolerance mechanism was studied in the present study. The function of mutated genes in F211 was identified by functional complementation experiments, revealing that the improved furfural tolerance was resulted from the C493T mutation of the ZCP4_0270 gene promoting cell flocculation and the mutation (G1075A)/downregulation of ZCP4_0970. Comparative transcriptome analysis revealed 139 differentially expressed genes between F211 and the control, CP4, in response to furfural stress. In addition, the reliability of the RNA-Seq data was also confirmed. The potential tolerance mechanism was further demonstrated by functional identification of tolerance genes as follows: (I) some upregulated or downregulated genes increase the levels of NAD(P)H, which is involved in the reduction of furfural to less toxic furfuryl alcohol, thus accelerating the detoxification of furfural; (II) the mutated ZCP4_0270 and upregulated cellulose synthetase gene (ZCP4_0241 and ZCP4_0242) increased flocculation to resist furfural stress; (III) upregulated molecular chaperone genes promote protein synthesis and repair stress-damaged proteins; and (IV) transporter genes ZCP4_1623–1,625 and ZCP4_1702–1703 were downregulated, saving energy for cell growth. The furfural-tolerant mechanism and corresponding functional genes were revealed, which provides a theoretical basis for developing robust chassis strains for synthetic biology efforts.

## Introduction

Lignocellulosic biomass is an abundant renewable and sustainable resource used to produce biofuels and high-value chemicals (Yang et al., 2020). A major hindrance in converting lignocellulose to ethanol is the recalcitrance of biomass degradation, and pretreatment processes are thus required to release fermentable sugars (Moreno et al., 2015). Furan aldehyde (furfural and hydroxymethyl furfural), phenolic chlorine compounds, and organic acids are the major inhibitors formed during the pretreatment process, which retards microbial fermentation and cellulose/hemicellulose hydrolysis (Yang et al., 2014). Therefore, breeding of inhibitor-tolerant strains and investigation of tolerance mechanisms have attracted a great deal of attention and providing strains and a theoretical basis for green bio-manufacturing (He et al., 2014; Pereira et al., 2020).

*Zymomonas mobilis*, a facultative anaerobic gram-negative bacterium that naturally produces ethanol, has excellent characteristics, such as a high specific rate of sugar uptake and high theoretical ethanol yield (Wang et al., 2018). It is also a promising chassis strain for the production of high value-added products from lignocellulosic biomass (He et al., 2014). *Zymomonas mobilis* detoxifies phenolic aldehydes to less toxic alcohols and exhibits stronger tolerance to phenolic acids than *Saccharomyces cerevisiae* (Gu et al., 2015). Nevertheless, aldehydes, especially furfural, tend to be more toxic to *Z. mobilis* than organic acids; therefore, the development of high furfural-tolerant *Z. mobilis* is crucial for the utilization of lignocellulose (Franden et al., 2009).

High furfural-tolerant *Z. mobilis* strains have been developed by directed evolution. For instance, ZMF3-3 (Shui et al., 2015) obtained by adaptive laboratory evolution (ALE) and ZM4-MF2 (Tan et al., 2015) developed from transcriptional engineering was able to tolerate 3g/L furfural. In our previous study, the strain F211 (Huang et al., 2018), obtained through error-prone PCR-based whole genome shuffling, could tolerate up to 3.5g/L of furfural. A rational design was also used to obtain furfural-tolerant strains. Overexpression of the ZMO1771 gene encoding alcohol dehydrogenase improved furfural tolerance by accelerating the reduction of furfural (Wang et al., 2017). Co-expression of ZMO1771 and *udhA* genes, encoding a transhydrogenase catalyzing the interconversion of NADH and NADPH, can maintain the balance between NAD(P)H/NADP^+^, resulting in a more effective furfural tolerance (Wang et al., 2017).

Under furfural stress, the growth and survival of *Z. mobilis* were inhibited, and the specific production rate of ethanol was correspondingly reduced (Franden et al., 2009; Nouri et al., 2020). In addition, furfural also inhibits the synthesis of various proteins, causes DNA damage, and leads to the differential expression of genes involved in membrane and cell wall biogenesis, transcriptional regulators, and energy metabolism (He et al., 2012; Yang et al., 2020). *Zymomonas mobilis* may respond to stress in the following ways: (1) conversion of furfural to furfuryl alcohol reduces toxicity (Wang et al., 2017); (2) the genes related to macromolecule synthesis are upregulated to resist furfural stress (Miller et al., 2009; Yang et al., 2020); and (3) transcription factors regulate the expression of multiple genes in response to stress (He et al., 2012; Nouri et al., 2020). However, the defined roles of these corresponding genes are not yet known.

In summary, there is still a lack of comprehensive understanding of furfural tolerance. The high furfural tolerance mutant strain F211 was obtained in an earlier study, and single nucleotide polymorphisms (SNPs) between F211 and control strain CP4 were identified by genome resequencing of F211 (Huang et al., 2018); in this study, functional identification of the mutations and RNA-Seq was therefore performed to investigate the genotypic changes associated with furfural tolerance and reveal the molecular mechanism responsible for the improved furfural tolerance in F211.

## Materials and Methods

### Bacterial Strain and Culture Conditions

The wild-type strain *Z. mobilis* CP4 was purchased from the China Center for Type Culture Collection (CCTTCC; Accession NO.CICC10232). F211 is a strain of *Z. mobilis* with high furfural tolerance obtained in the early stage of our laboratory (Huang et al., 2018). *Zymomonas mobilis* was cultured in Rich Medium (10g/L yeast extract, 2g/L KH_2_PO_4_, 20g/L glucose, and different concentration of furfural as required) at 30°C without shaking.

### Construction of Deletion and Overexpression Strains

Nine deletion strains (Supplementary Table S1) were constructed by homologous recombination as described in our previous study with some modification (Wang et al., 2013; Huang et al., 2018). The left and right homologous arm DNA fragments of the target gene were ligated to flank both sides of a chloramphenicol resistance gene, generating the homologous recombinant fragments (Section 1.1 of Supplementary Materials and Methods). The homologous recombinant fragments were ligated into the *Sph*I and *Sac*II sites of pUC19 to yield the plasmid pT-gene (Supplementary Table S1) by using the Gibson assembly (GA; Gibson et al., 2009). The target plasmids were then transformed into CP4 competent cells by electroporation (Ma et al., 2012). The target gene was replaced with the chloramphenicol resistance gene by homologous recombination using its native RecA recombinase (Supplementary Figure S1; Savvides et al., 2000; Huang et al., 2018). Transformants were screened on RM agar plates with 50μg/ml chloramphenicol. Positive single colonies were confirmed by PCR using the appropriate geneGF/geneGR, geneGF/T-CM-R, and T-CM-F/geneGR (Supplementary Table S2) primer pairs (Supplementary Materials and Methods).

Overexpression vectors were constructed by ligating the target wild-type gene or its mutated gene into multiple clone sites of pEZ15Asp (pE)/pHW20a (pH) vector. The target genes were amplified from the genomes of CP4 and F211 using the primers gene OF/OR (Supplementary Table S3), and the P*_gap_* promoter (Yang et al., 2019) was amplified from the genome of CP4 using the primers pgap-F/pgap-R (Supplementary Table S3). Then, the two fragments were fused by overlap-extension PCR using the primers pgap-F/gene-OR. The fusion fragment was inserted into the *Pst*I and *Eco*RI sites of pE/pH to yield the plasmid pE-gene/pH-gene by using the aforementioned GA (Gibson et al., 2009). Positive clones were further verified by DNA sequencing. The correctly sequenced recombinant plasmids were transformed into CP4 competent cells by electroporation. The target plasmids were confirmed by PCR using the appropriate pEZ-F/pEZ-R (Supplementary Table S3) primer pairs.

### Furfural Tolerance Assay

Strain was precultured in RM at 30°C to the late exponential stage, and the cultures were concentrated to an OD_600_ of 20 by centrifugation. The concentrated cells were inoculated into 100ml RM medium containing 3.5g/L furfural with an initial OD_600_ of 0.2, and the OD_600_ value was measured every 6h to detect the growth of the cells for the evaluation of furfural tolerance. The degradation rate of furfural was calculated by the following formula:


$$\mathrm{The degradation rate of furfural}=\frac{C_{initial}-C_{final}}{T}$$

where “T” indicated the fermentation time, and “C_initial_” and “C_final_” indicated “initial medium furfural concentration” and “final medium furfural concentration,” respectively. All experiments were performed in triplicates, and statistical differences were analyzed using a one-way analysis of variance (ANOVA). *p<0.05 was considered statistically significant, and **p<0.01 was considered extremely significant.

### Flocculation Measurement

Strains were cultured in RM medium containing 3g/L furfural for 36h, and 10μl of the culture was taken to observe the flocculation under a light microscope (Olympus BX51, Japan). Five milliliter culture of each strain was collected and was shaken vigorously for 15s and then rested for 5min. Suspension of 1ml was sampled from the upper section to measure OD_600_ and quantify the non-flocculating cells (*Cf*), and 1.2units of cellulase (Novozymes, Denmark) was added to the remaining culture, which was incubated at 50°C for complete de-flocculation of the bacterial flocs to measure the concentration of total cells (Ct). The flocculation efficiency of *Z. mobilis* was calculated based on the formula:

$R\%=\left(1-\frac{\mathrm{Cf}}{\mathrm{Ct}}\right)$×100 (Xia et al., 2018).

### RNA-Seq Transcriptomic Analysis

Strain F211 and CP4 were cultured statically in RM medium with 3g/L furfural. The cultures were collected by centrifugation when the OD_600_ reached 0.8–1.4, and the cells were washed with PBS buffer (pH=7.4, 137mM NaCl, 2.7mM KCl, 10mM Na_2_HPO_4_·12H_2_O, and 1.4mM KH_2_PO_4_). The cells were collected by centrifugation and freeze in liquid nitrogen for 1h. The samples were submitted to Novogene (China, Beijing) for RNA-Seq.

The integrity of the extracted RNA was tested using the Agilent Bioanalyzer 2,100 system (Agilent Technologies, CA, United States). cDNA library preparation and sequencing were conducted by the Novogene Technology Company in Beijing, China. The clean reads were obtained by sequencing and mapped to the *Z. mobilis* CP4 genome sequence (GenBank: CP006818.1) using Bowtie2-2.2.3. Differentially expressed genes (DEGs) between the two samples were identified using the DESeq package (1.20.0), and |log2(FoldChange)|>1 and q-value<0.005 were used as the threshold to identify significant differences in gene expression analysis of Gene Ontology (GO), which assigns genes into functional categories, was performed using the GO R packages. KOBAS software could perform KEGG enrichment analysis on differentially expressed genes to determine the metabolic pathways of genes.

### Real-Time Quantitative Reverse Transcription PCR Analysis

The expression levels of 22 DEGs representing different functional categories were assessed by real-time quantitative reverse transcription PCR (qRT-PCR) to validate the reliability of the RNA-Seq data. The culture conditions of CP4 and F211 were the same as the RNA-Seq experiment. When the strains concentration reached OD_600_=0.8–1.4, about 10^8^ cells were collected from each strain, and the cells are treated in liquid nitrogen for 5min. Subsequently, the bacterial cells were ground in a mortar pre-cooled with liquid nitrogen, and the powder was transferred to 100μl TE buffer (containing 400μg/ml lysozyme), mixed, and placed at room temperature for 4min to dissolve the cell wall. RNA was extracted with TRIzol reagent (Invitrogen, Carlsbad, CA, United States) according to the manufacturer’s instructions.

DNase (RQ1 RNase-Free DNase, Promega, United States) was added to the RNA samples to remove genomic DNA. The PCR reaction with primer pair 16SrRNA-F/16SrRNA-R was performed on the total RNA in order to check for genomic DNA contamination. No product was observed, demonstrating that the sample had no genomic DNA contaminants and was suitable for qRT-PCR assay. Subsequently, cDNA was synthesized using iScript cDNA Synthesis Kit (Bio-Rad) with the purified total RNA of CP4 or F211 as a template following the manufacturer’s instructions. Real-time qRT-PCR experiments were performed using CFX96 Real-Time System (Bio-Rad). Each reaction contained 2μl of diluted (1/10) cDNA, Taq SYBR Green qPCR Premix (Yugong Biolabs Inc., Jiangsu, China) and the corresponding primer pairs (Supplementary Table S4). The 16S rRNA gene was used as a reference to normalize the qRT-PCR data. The expression level of each gene was calculated according to the 2(-Delta Delta C(T)) method (Livak and Schmittgen, 2001).

## Results and Discussion

### Functional Identification of Mutated Genes in F211

Eleven SNPs (single nucleotide polymorphisms) were found in high furfural-tolerant F211 compared with the sequence (GenBank No. CP006818) of CP4 from Agricultural Research Service Culture Collection (NRRL No. B-14023; Huang et al., 2018). The initial strain CP4 (CICC 10132) of F211 and the reference CP4 may have different SNPs. Therefore, in this study, except for the three synonymous mutated genes of ZCP4_0096, ZCP4_0593, and ZCP4_1887, seven missense mutated genes and one nonsense mutated gene of F211, along with the starting strain, CP4, were PCR-amplified and sequenced. F211 and CP4 shared consistent sequences of ZCP4_1431, ZCP4_1,616, ZCP4_0244, and ZCP4_0525. The ZCP4_1919, ZCP4_1419, and ZCP4_0270 genes in F211 carried missense mutations in G335A, C85T, and C493T, resulting in a substitution of V119I, G29S, and G165S, respectively. The mutation of ZCP4_0970 (1075G>A) converted a glutamine codon to a stop codon (Q359X), producing a truncated protein lacking 153 amino acids at the C-terminal end (Table 1). Therefore, these four genes were selected for functional studies related to furfural tolerance.

**Table 1:** Mutations identified in the F211 strain.

Gene	Function	Mutation	Effect
ZCP4_0270	Diguanylate cyclase/phosphodiesterase	C493T	G165S
ZCP4_0970	Type I secretion outer membrane protein, TolC family	G1075A	Q359X
ZCP4_1419	Surface lipoprotein	C85T	G29S
ZCP4_1919	A C-terminal OMP(outer membrane protein) domain	G355A	V119I

*X, Termination codon*.

**Figure 1:**
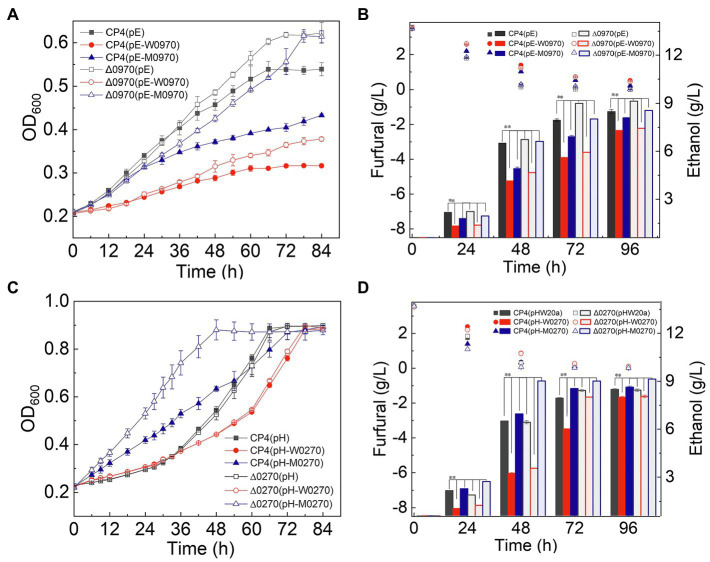
Growth and fermentation of the functionally complementary strains of genes ZCP4_0270 and ZCP4_0970. Panel (A) and (C) indicated that the growth curves of engineered strains of ZCP4_0970 and ZCP4_0270 genes, respectively. The furfural (scatter plot) and ethanol (bar) concentration in RM medium of engineered strains of ZCP4_0970 and ZCP4_0270 genes were shown in Panel (B) and (D), respectively.

The deletion and overexpression strains of these genes were constructed to study furfural tolerance. In RM medium containing 3.5g/L furfural, the deletion and overexpression strains of ZCP4_1419 and ZCP4_1919 genes did not show obvious growth differences (Supplementary Figures S3A,B), indicating that these two genes have no effect on furfural tolerance. The ZCP4_0970 gene encodes the TolC family protein of the bacterial type I secretion system, transporting proteins to the extracellular space coupled with ATP consumption (Delepelaire, 2004). The deletion strain Δ0970 (pE) showed a 13 and 7.6% increase in the maximum cell density and ethanol production, respectively, compared to the control CP4 (pE) (Figures 1A,B). In contrast, the overexpression strain CP4 (pE-W0970) had a 47 and 13.9% decrease in the maximum cell density and ethanol production, respectively, compared to the control CP4 (pE) (Figures 1A,B). The results showed that the downregulation of ZCP4_0970 can increase the furfural tolerance of strains, which indicates that the G1075A mutation in the ZCP4_0970 gene resulted in functional inactivation or attenuation, improving furfural tolerance.

Furfural destroys the tertiary structure of proteins and inhibits protein synthesis (He et al., 2012; Todhanakasem et al., 2014). Tolc inactivation leads to defects in the secretion system and the increase in ATP levels (Delepelaire, 2004), thereby providing more energy for cell growth and increasing furfural tolerance. Interesting, the deletion of the ZCP4_0970 gene destroys the efflux system, also yielding a reducing tetracycline resistance (Delepelaire, 2004; De Cristóbal et al., 2006), which causes the inability of transformants to grow on the tetracycline selection plates. And the original initial pHW20a plasmid containing tetracycline resistance gene (Dong et al., 2011) could not be transformed successfully into Δ0970 strain. So, the plasmid pEZ15Asp with an ampicillin resistance gene (Yang et al., 2016) was used as a starting vector for construction of wild-type and mutated ZCP4_0970 gene overexpression vectors.

ZCP4_0270 encodes a bifunctional protein with diguanylate cyclase (DGC)/phosphodiesterase (PDE), which synthesizes/degrades bis-(39–59)-cyclic dimeric guanosine monophosphate (c-di-GMP). The deletion strain Δ0270 (pH) and overexpression strain Δ0270 (pH-W0270) did not show significant growth differences compared to those of the control strains CP4 (pH) and Δ0270 (pE-W0270) (Figures 1C,D), indicating that deletion or overexpression of the native ZCP4_0270 gene did not affect furfural tolerance. The specific growth rate, degradation rate of furfural in the medium (0–48h), and ethanol production of the mutated gene overexpression strain Δ0270 (pH-M0270) were 62, 8.3, and 38.5% higher than those of the control strain Δ0270 (pE-M0270) (Figures 1C,D), respectively, indicating that the C493T mutation of the ZCP4_0270 gene can promote the degradation of furfural, thereby improving furfural tolerance.

**Figure 2:**
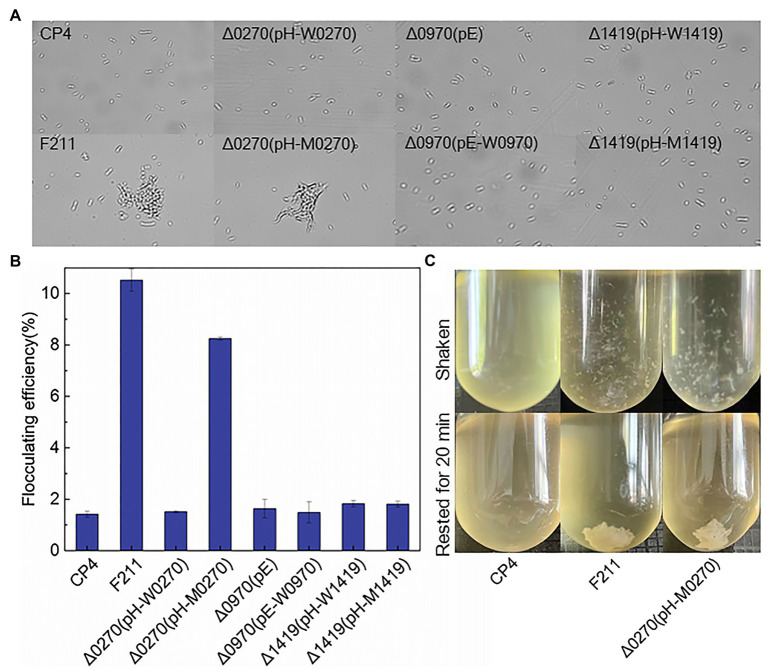
Forces in *Zymomonas mobilis* flocculation. **(A)** Flocculent microscopic image of recombinant strains. **(B)** Flocculation rate. **(C)** Flocculation phenotype.

Flocculated yeast showed a higher tolerance to furfural than free yeast (Westman, 2014). The increased c-di-GMP, which is regulated by PDEs, can promote the flocculation of *Z. mobilis* (Tan et al., 2014). Therefore, the mutated ZCP4_0270 may improve furfural tolerance by promoting flocculation, and thus, the flocculation of F211 and Δ0270 (pH-M0270) was investigated. Flocculation was observed in both strains (Figures 2A,C), and the flocculation rates of F211 and Δ0270 (pH-M0270) were 600 and 470% higher than that of CP4, respectively (Figure 2B). The flocculation rate of the wild-type ZCP4_0270 gene overexpressing strain Δ0270 (pH-W0270) and the engineered strains of ZCP4_0970 and ZCP4_1419 were similar to those of the control CP4 (Figure 2B). These results show that the mutated ZCP4_0270 gene in F211 causes flocculation, resulting in an improved furfural tolerance.

### Transcriptome Analysis of F211 and CP4 Under Furfural Stress

The bifunctional DGCs/PDEs encoded by the ZCP4_0270 gene can regulate the levels of the second messenger c-di-GMP, which coordinates diverse aspects of bacterial growth and behavior, including motility, virulence, biofilm formation, and cell cycle progression (Jenal et al., 2017). Therefore, enhanced tolerance by the mutated ZCP4_0270 also resulted in a change in the physiological, biochemical, and expression levels of some genes. Consequently, RNA-Seq was performed to analyze the transcriptome differences between F211 and CP4 under furfural stress and to explore the potential furfural tolerance mechanism.

A total of 139 DEGs were identified in F211 compared with the control, with 77 and 62 genes being upregulated and downregulated, respectively. The expression levels of the three missense genes in F211 did not change significantly, indicating that its enhanced tolerance was due to the changed protein function caused by the mutated ZCP4_0270 and ZCP4_0970 genes, rather than the changed transcript abundance. DEGs were subjected to GO enrichment analysis. The five GO clusters with the highest enrichment were as follows: “localization,” “transport,” “establishment of localization,” “cofactor binding,” and “response to stimulus” (Supplementary Figure S4A). The DEGs were enriched in 39 pathways, found using a Kyoto Encyclopedia of Genes and Genomes (KEGG) analysis, 27 of which were different from those previously reported (He et al., 2012; Yang et al., 2020). The significant KEGG terms were primarily associated with microbial metabolism in diverse environments, biosynthesis of secondary metabolites and antibiotics, bacterial chemotaxis, and sulfur metabolism (Supplementary Figure S4B).

**Figure 3:**
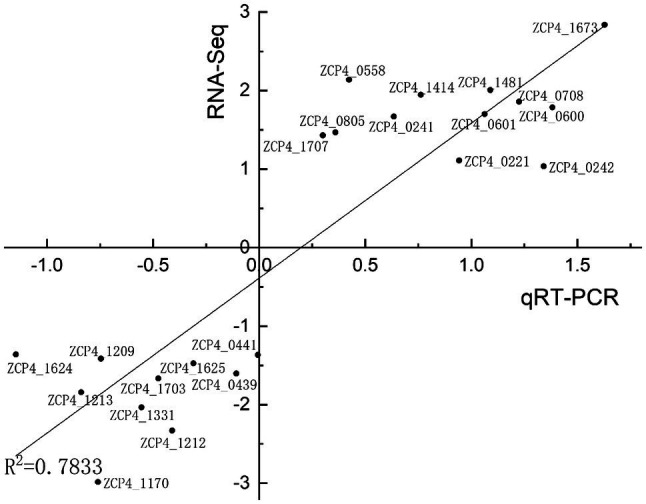
Correlation between RNA-seq and qRT-PCR results for RNA-seq data verification. The gene expression ratios of RNA-seq and qRT-PCR data for the genes in Table 2 were log transformed (base 2), and the RNA-seq log2 ratio values were plotted against the qRT-PCR log2 values.

We screened 22 DEGs representing different GO clusters from 139 DEGs (Table 2) for qRT-PCR verification. Based on the qRT-PCR results, the expression trends of these candidate genes were consistent with the RNA-Seq data (Figure 3). A comparison of the two methods indicated a high level of concordance (*R*^2^=0.7833), indicating that the RNA-Seq data were reliable.

**Table 2:** The differentially expressed genes used for qRT-PCR verification.

Functional group and gene	Description	Log2 fold change in RNA-Seq	Log2 fold change in qRT-PCR
**Transporter**			
ZCP4_0708	MetI-like ABC transporter transmembrane protein	1.858	1.225
ZCP4_1,170	Anion permease, Sulfite exporter TauE/SafE	−2.986	−0.759
ZCP4_1331	Alginate export	−2.033	−0.554
ZCP4_1481	Aspartate-alanine antiporter	2.006	1.090
ZCP4_1624	Multidrug efflux pump subunit AcrB	−1.359	−1.147
ZCP4_1,625	Efflux transporter outer membrane factor lipoprotein, NodT family	−1.474	−0.310
ZCP4_1703	NodT family RND efflux system outer membrane lipoprotein	−1.665	−0.475
**Stress related**			
ZCP4_0558	General stress protein CsbD	2.138	0.424
ZCP4_0600	YfdX protein, putative chaperone	1.785	1.382
ZCP4_0601	OsmC family protein	1.701	1.062
ZCP4_1707	ATP-dependent chaperone ClpB	1.467	0.359
**Metabolism**			
ZCP4_1209	Sulfite reductase subunit beta, CysI	−1.414	−0.745
ZCP4_1212	Sulfate adenylyltransferase subunit 1, CysD	−2.331	−0.410
ZCP4_1213	Sulfate adenylyltransferase subunit 2, CysN	−1.842	−0.839
ZCP4_1,414	Succinate-semialdehyde dehydrogenase	1.945	0.761
ZCP4_1673	Acyltransferase of alpha/beta superfamily	2.836	1.629
**Membrane related**			
ZCP4_0241	Cellulose synthase operon C domain protein, BscC	1.670	0.635
ZCP4_0242	Cellulose synthase subunit B, BscB	1.035	1.341
ZCP4_0439	Squalene-hopene cyclase	−1.604	−0.108
ZCP4_0441	Squalene synthase HpnD	−1.367	−0.006
**Regulator**			
ZCP4_0221	AsnC family transcriptional regulator	1.109	0.943
ZCP4_0805	Transcriptional regulator	1.431	0.300

**Figure 4:**
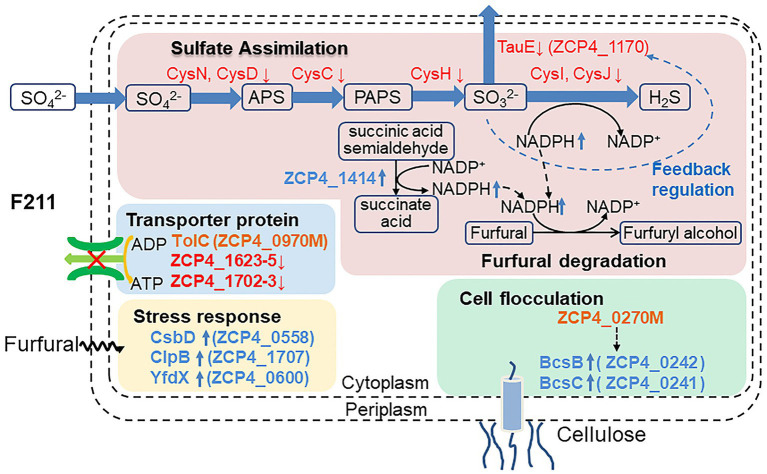
Potential furfural tolerance mechanism of *Z. mobilis*.

### Furfural Tolerance Mechanism by Functional Analysis of DEGs

#### Increased Intracellular NAD(P)H Levels Resist Furfural Stress

**Figure 5:**
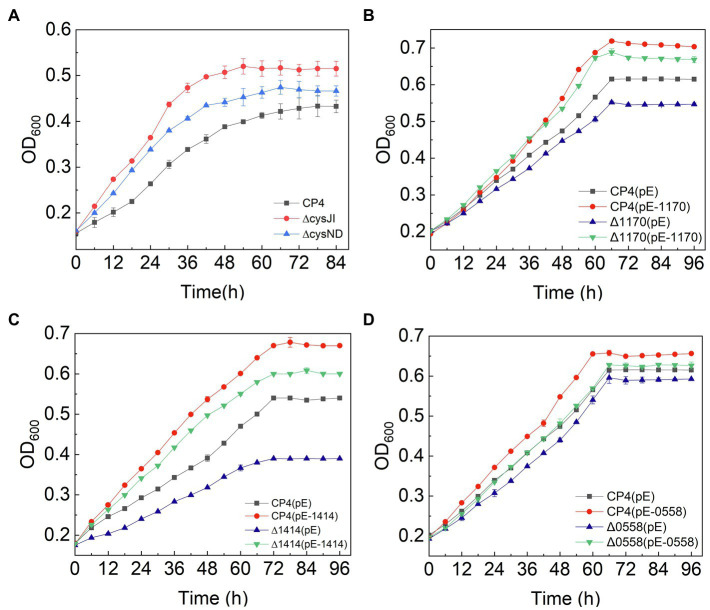
Growth curves of engineered strains of *CysJI*, *CysND*, ZCP4_1,170, ZCP4_1,414, and ZCP4_0558 genes.

The sulfur assimilation operon *CysJIH* (ZCP4_1208–1,210) and *CysGDNC* (ZCP4_1211–1,214) were significantly downregulated in F211 in response to furfural stress (Table 2). Deletion strains of these genes were engineered to investigate their furfural tolerance. The cell densities of the deletion strains ΔCysJI and ΔCysND were increased by 20 and 9% compared to those of CP4 (Figure 5A), respectively, showing that the deletion of these genes could improve the furfural tolerance. The enzymes encoded by *CysND* and *CysJI*(*G*) genes involved in cysteine synthesis coupled with ATP and NAD(P)H consumption (Figure 4; Miller et al., 2009; Takagi, 2019). And the conversion of NAD(P)H to NAD(P)^+^ was coupled with reduction in furfural to furfuryl alcohol (Boopathy, 2009; Yi et al., 2015). The concentration of NADH of the ΔCysND and ΔCysJI was 35.7-fold and 44.6-fold higher than that of the control CP4 in RM without furfural (Supplementary Figure S5A), respectively, indicating that deletion of these genes can reduce NADH consumption. While the NADH/NAD^+^ ratio was decreased by 49.5 and 34.5% for ΔCysND and ΔCysJI under furfural stress, respectively, compared with non-stress condition (Supplementary Figure S5B), demonstrating that the deletion strains consumed more NADH under furfural stress. Thereby, the downregulation of these genes could reduce the consumption of NADH, and the saved NADH may be used for the conversion of furfural to furfural alcohol, thus enhancing the detoxification of furfural. The damaged proteins and DNA were correspondingly reduced.

Interestingly, overexpression of the cysteine synthase operon *CysCNDG* (ZMO0003–0006) contributed to the production of cysteine, replenishing proteins damaged by furfural, thus improving the furfural tolerance of 8b (Miller et al., 2009; Yang et al., 2020). The destruction of sulfur assimilation pathway can reduce the synthesis of cysteine, a deficiency of amino acids for growth (Miller et al., 2009) and response stress. Therefore, the furfural tolerance characteristics of ΔCysJI and ΔCysND strain may be counteracted partially by the Cys deficiency. In our study, the downregulation of the *CysCNDG* operon in F211 may be caused by a mutation in the ZCP4_0270 gene, which regulates the content of c-di-GMP, causing physiological and biochemical changes such as cell flocculation (Tan et al., 2014).

The ZCP4_1,170 gene, encoding the efflux pump protein TauE, involved in efflux sulfite to the periplasmic space, is downregulated 3.0-fold under furfural stress. The overexpression and deletion strains of this gene were constructed to demonstrate their function. Compared with the control strain CP4 (pE), the cell density of the overexpression strain CP4 (pE-1,170) and deletion strain Δ1170 (pE) was increased and decreased by 21 and 22% (Figure 5B), respectively, showing that its upregulated expression improved furfural tolerance. Conversely, the expression of the ZCP4_1,170 gene is downregulated in furfural-tolerant F211 (Table 2), which results from feedback regulation by sulfate assimilation genes. The downregulation of *CysH* and *CysDNC* genes reduces sulfite synthesis and this may have corresponded with less TauE being needed; therefore, the genes were feedback downregulated.

The expression of the ZCP4_1,414 gene encoding succinate dehydrogenase was upregulated by 1.9-fold in F211 (Table 2). The enzyme catalyzes the conversion of succinic acid semialdehyde to succinate acid and concomitantly reduces NAD(P)^+^ to NAD(P)H. The overexpression strains CP4 (pE-1,414) and Δ1414 (pE-1,414) of this gene showed a 24 and 11% increase (Figure 5C) in cell density compared with the control strains CP4 (pE) and Δ1414 (pE), indicating that its upregulation improved furfural tolerance. Furthermore, its upregulation can accelerate the regeneration of NAD(P)H and provide more reducing activity for furfural detoxification, thereby resulting in enhanced tolerance.

#### Responses of Molecular Chaperones and Stress Proteins to Furfural Stress

The ZCP4_0558 gene encoding a universal stress protein CsbD, molecular chaperone *yfdX* (ZCP4_0600), and *clpB* (ZCP4_1707), was significantly upregulated in F211 under furfural stress (Table 2). The overexpression and deletion strains of the ZCP4_0558 gene were constructed to study the effect of its expression levels on furfural tolerance. The specific growth rate of the overexpression strain CP4 (pE-0558) and the deletion strain Δ0558 (pE) were increased and decreased by 27.6 and 9.5%, respectively, compared with the control strain CP4 (pE) (Figure 5D), indicating that its upregulation could improve furfural tolerance. The *csbD* from *Methylocystis* was upregulated under stress of starvation, high heat, and acid (Han et al., 2017). CsbD from *Bacillus coagulans* also showed upregulated expression in response to furfural stress (van der Pol et al., 2016). To our knowledge, this study provides the first evidence that its upregulation can improve the furfural tolerance of *Z. mobilis*. Chaperones play important roles in cell survival, and they can promote the folding of peptide chains and repair stress-damaged proteins, thus ensuring protein homeostasis in cells (Liu et al., 2016; Ghazaei, 2017). Furfural inhibits synthesis of various proteins and affects cell growth and survival (Franden et al., 2009; He et al., 2012). Therefore, the upregulated expression of molecular chaperones in F211 may promote protein synthesis, reduce damage to cells, and improve furfural tolerance.

#### Cell Flocculation

Two genes (ZCP4_0241 and ZCP4_0242) related to cellulose synthesis were upregulated in F211 (Table 2). The increased cellulose content on the cell surface contributes to cell flocculation (Jeon et al., 2012; Xia et al., 2018). The c-di-GMP, regulated by PDEs encoded by the ZCP_0270 gene, is an activator of cellulose synthesis (Xia et al., 2018); therefore, it may be because the mutated ZCP4_0270 gene may increase the activity of diguanylate cyclase, enhancing the content of c-di-GMP and activating cellulose synthesis, resulting in cell flocculation to resist stress. In addition, the chemotaxis (ZCP4_1147 and ZCP4_1150) and flagella (ZCP4_0645) genes were downregulated in F211 (Supplementary Table S5), which may reduce cell motility and facilitate cell flocculation, thus improving furfural tolerance (Jeon et al., 2012; Xia et al., 2018). A stronger flocculation ability was observed in F211 compared to CP4 (Figure 2A), demonstrating the potential furfural tolerance mechanism.

#### Reduce Energy Consumption of Efflux Pump

The ATP-binding cassette (ABC) transporter family (ZCP4_1623, ZCP4_1624, and ZCP4_1,625) genes and the resistance-nodulation-division (RND) family (ZCP4_1702 and ZCP4_1703) genes were downregulated in F211 (Table 2). They consume ATP to export lipids, amino acids, heavy metals, etc., to the outside of the cell (Saier and Paulsen, 2001). Deletion of an operon encoding the RND efflux system could improve the furfural tolerance of *Z. mobilis* ZM4 (Yang et al., 2020). The downregulation of these efflux pump genes may reduce the energy consumption of cells, providing more energy for cell growth and increasing furfural tolerance. In future, corresponding functional verification experiment would be performed to consolidate the current discussion.

## Conclusion

In conclusion, F211 resists furfural stress by accelerating the detoxification of furfural, improving cell flocculation, enhancing emergency response, and reducing energy consumption. This study lays the foundation for the rational design of high furfural tolerance strains, which would accelerate the industrialization process of lignocellulosic ethanol.

## Data Availability Statement

The RNA-seq datasets for this study can be found in the NCBI under accession number PRJNA743578.

## Author Contributions

YM conceived of the project, analyzed the data, supervised the study, and revised the manuscript. MH supervised the study and revised the manuscript. DH and ZW performed the experiments and drafted the manuscript. All authors contributed to the article and approved the submitted version.

## Funding

This work was supported by the Tianjin Science & Technology Council (18JCYBJC24200), the National Natural Sciences Foundation of China (32070036), the Elite Program and Basic Research Program of Chinese Academy of Agricultural Sciences, and the Key Laboratory of Development and Application of Rural Renewable Energy, and the Ministry of Agriculture and Rural Affairs, China (2019003).

## Conflict of Interest

The authors declare that the research was conducted in the absence of any commercial or financial relationships that could be construed as a potential conflict of interest.

## Publisher’s Note

All claims expressed in this article are solely those of the authors and do not necessarily represent those of their affiliated organizations, or those of the publisher, the editors and the reviewers. Any product that may be evaluated in this article, or claim that may be made by its manufacturer, is not guaranteed or endorsed by the publisher.
