# Supplementary material for: Functional Gene Identification and Corresponding Tolerant Mechanism of High Furfural-Tolerant Zymomonas mobilis Strain F211
Source: Front Microbiol. 2021 Nov 11;12:736583. doi: 10.3389/fmicb.2021.736583 (PMC8631904; doi:10.3389/fmicb.2021.736583)
Supplement: Supplementary file 1 [file Data_Sheet_1.docx]

Supplementary Material

# Supplementary Material and methods

**1.1 Construction of deletion mutants**

The left and right homology arms of the target genes were amplified from the CP4 genome using the primer pairs geneLarmF/R and geneRarmF/R (Table S2), and the chloramphenicol resistance cassettes flanked by homologous sequence of the left and right homology arms were amplified from plasmid pKD3 by PCR using primers cm-gene-F/R (Table S2). The three resulting DNA fragments were ligated into the *Sph*I and *Sac*II sites of pUC19 to yield the plasmid pT-gene (Table S1) by using the Gibson assembly (GA). The plasmids were then electrotransformed into competent CP4 cells, and the transformants were screened on RM plates with 50 μg/mL chloramphenicol at 30 ℃. (Figure S1). Several transformants were picked up for PCR verification.

The Colony PCR was performed to detect the deletion of target gene as follows: three primer pairs geneGF/T-CM-R, T-CM-F/geneGR and geneGF/geneGR were used to identify the disruption of the target genes according to the published protocol (Datsenko et al. 2000; Agrawal et al. 2012). The primers geneGF and T-CM-R were used to identify the junction between left homologous arm and Cm^R^, and primer pair T-CM-F/geneGR were used to amplify the junction between Cm^R^ and right homologous arm. Primer pair geneGF/geneGR was used to verify simultaneous loss of the target gene and gain of a novel DNA fragment (the Cm^R^) (Figure S2). The PCR products were then sequenced to confirm the deletion of the target gene. The deletion mutants obtained were shown in Table S1.

**1.2 Identification and confirmation of SNPs in F211 and CP4**

The cassettes of the wildtype and mutated genes were amplified from F211(Huang et al., 2018) and CP4 (CICC 10132) genome using primers listed in Table S6. The reaction system was as followed: 10 ng of genome, 10 pmol each of upstream and downstream primers and 25 μL of 2 × Phanta max master mix (Vazyme, China) in a final volume of 50. PCR was performed using a standard program: 94℃ predenaturation for 3 min; 30 cycles of 15s denaturation at 94℃, 15 s annealing at 45℃ , 1–3 min extention at 72℃ and concluded with a 5 min final extension at 72℃. The extension time was calculated according to the expected product sizes (1 min/kb). The PCR products were purified by TIANgel Midi Purification Kit (TIANGEN, China), and then verified by DNA sequencing (Songon Biotech, Shanghai). The mutated genes are shown in Table 1.

## Determination of NADH and NAD^+^ concentrations

The CP4, ΔCysIJ and ΔCysDN strains were cultured in RM medium containing 0 or 3 g/L furfural. Five million cells were collected when the strain grew to late exponential stage. The pellet was resuspended in alkaline (acidic) extract, and prepared the crude solution, which was used to determine the concentration of NADH and NAD^+^ by the reduction method according to the manufacturer’s instructions (Coenzyme I NAD(H) assay kit, Solarbio, China).

# Supplementary Data

There are a large number of repetitive sequences in the gene structure of ZCP4_1919, which cannot be obtained by PCR amplification or synthesis. Therefore, only the deletion strain Δ1919 was obtained (Figure S1).

##
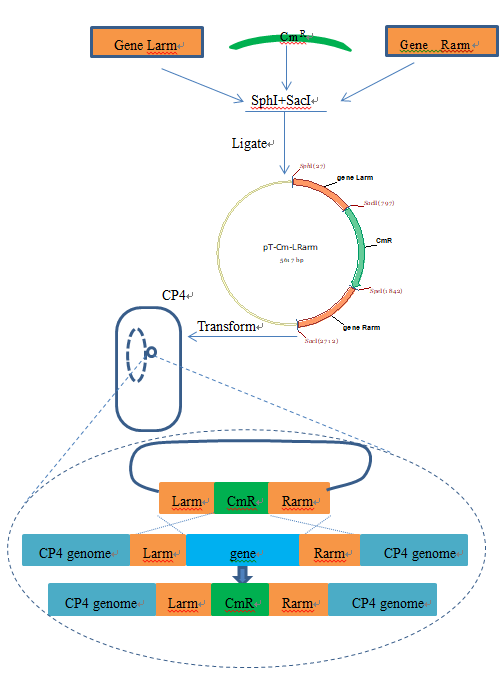
Supplementary Figure

**Supplementary Figure 1.** Construction of the deletion mutant strains


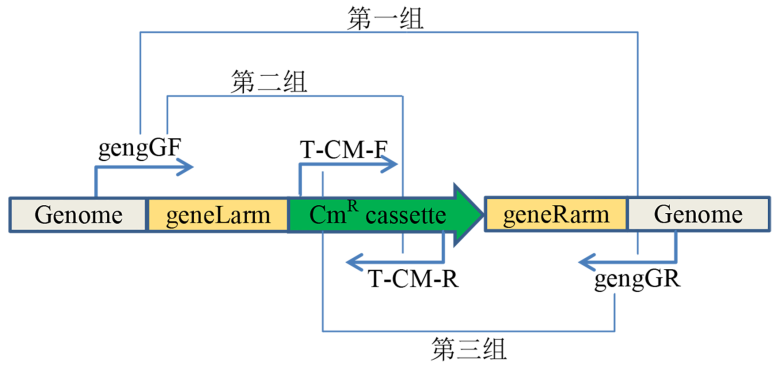


The first group

The second group

The third group

**Supplementary Figure 2.** The position of the primers for identifying the deletion mutant strains


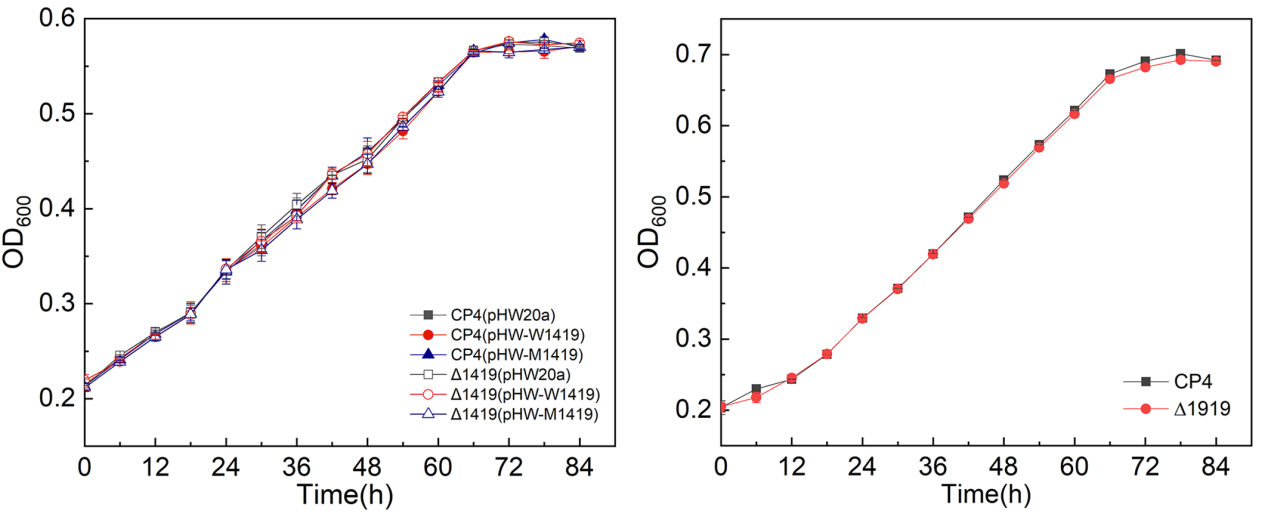


**Supplementary Figure 3.** Growth of ZCP4_1419 and ZCP4_1919 functional complementary strains with 3.5 g/L furfural stress.


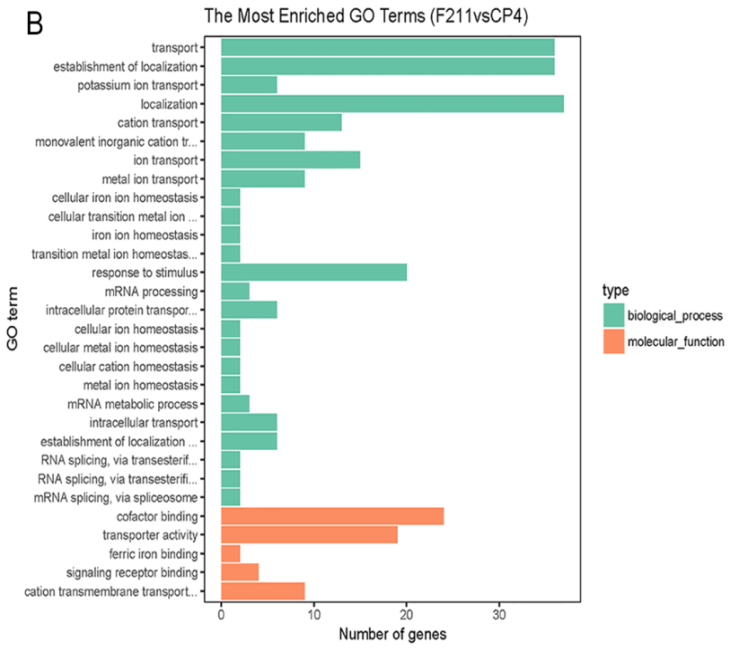

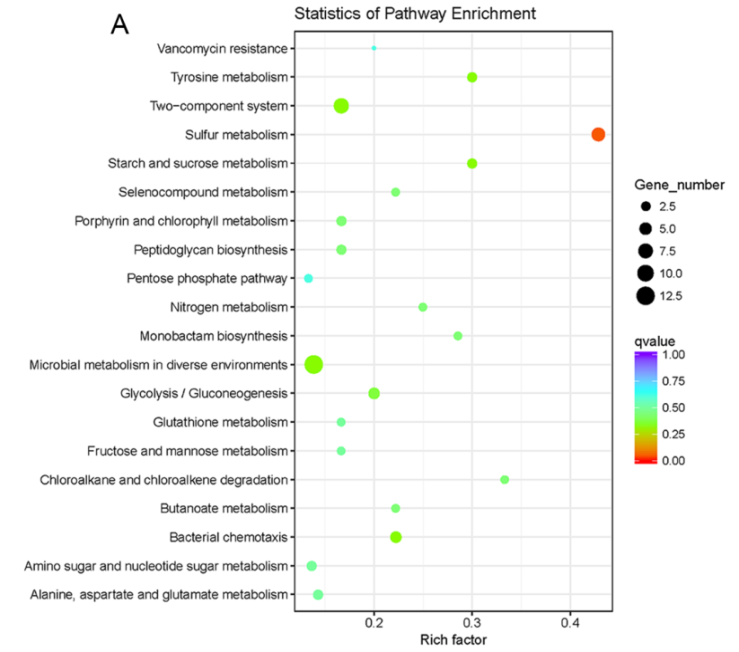


**Supplementary Figure 4.** GO cluster analysis and KEGG enrichment analysis of differentially expressed genes.







A B

**Supplementary Figure 5.** Measurements of NADH and NAD^+^ level. (A) Contents of intracellular NADH and NAD^+^; (B) Calculation of the NADH/NAD^+^ ratio.

## Supplementary Tables

**Table. S1** **Deletion strain and plasmids constructed in this study.**

| **Strains and plasmids** | **Genotype/relevant characteristics** |
| --- | --- |
| Strains  Δ1919 | CP4 △ZCP4_1919 |
| Δ1419 | CP4 △ZCP4_1419 |
| Δ0270 | CP4 △ZCP4_0270 |
| Δ0970 | CP4 △ZCP4_0270 |
| ΔcysJI | CP4 △*cysJ*△*cysI* |
| ΔcysND | CP4 △*cysN*△*cysD* |
| Δ1170 | CP4 △ZCP4_1170 |
| Δ1414 | CP4 △ZCP4_1414 |
| Δ0558 | CP4 △ZCP4_0558 |
| Plasmids |  |
| pT-1919 | plasmid pGEMT-easy containing achloramphenicol acetyltransferase expression cassette and the left and right homology arms of the ZCP4_1919 gene |
| pT-1419 | plasmid pGEMT-easy containing achloramphenicol acetyltransferase expression cassette and the left and right homology arms of the ZCP4_1419 gene |
| pT-0270 | plasmid pGEMT-easy containing achloramphenicol acetyltransferase expression cassette and the left and right homology arms of the ZCP4_0270 gene |
| pT-0970 | plasmid pGEMT-easy containing achloramphenicol acetyltransferase expression cassette and the left and right homology arms of the ZCP4_0970 gene |
| pT-cysJI | plasmid pGEMT-easy containing achloramphenicol acetyltransferase expression cassette and the left and right homology arms of the *cysIJ* gene |
| pT-cysND | plasmid pGEMT-easy containing achloramphenicol acetyltransferase expression cassette and the left and right homology arms of the *cysND* gene |
| pT-1170 | plasmid pGEMT-easy containing achloramphenicol acetyltransferase expression cassette and the left and right homology arms of the ZCP4_1170 gene |
| pT-1414 | plasmid pGEMT-easy containing achloramphenicol acetyltransferase expression cassette and the left and right homology arms of the ZCP4_1414 gene |
| pT-0558 | plasmid pGEMT-easy containing achloramphenicol acetyltransferase expression cassette and the left and right homology arms of the ZCP4_0558 gene |

**Table. S2 Primers in knockout experiments with the homologous recombination.**

| Primers | Sequence（5’-3’） |
| --- | --- |
| 0270LarmF | ATTAT*GCATGCCCGGG*ACAGATAAAGTAGAGGCCGCTGGAA |
| 0270LarmR | ATGAG*CCGCGGTACC*ATGAAGCGACAGCAGGATACCA |
| 0270RarmF | ACCGC*ACTAGT*CTCGAGGAAGTATCCGAAGATTTGTTGAGGG |
| 0270RarmR | AGTAT*GAGCTCGGATCC*TCAACAGGTAACGGACGGGAAAA |
| 0970LarmF | ATTAT*GCATGC*CCGGGGATGGCGTTATCCTGTAACGCT |
| 0970LarmR | ATGAG*CCGCGG*TACCAGGTTTCTGCTTGATTGACCGT |
| 0970RarmF | ACCGC*ACTAGT*CTCGAGATCATCGAAGATTTATCGCCCG |
| 0970RarmR | AGTAT*GAGCTC*GGATCCCCTCGGCTGATCCTGAATGAT |
| 1419LarmF | ATTAT*GCATGCCCGGG*GGTGGCCTTTTCTACCCTGTTATC |
| 1419LarmR | ATGAG*CCGCGG*ATTGGGCAATACCGGTACCAAC |
| 1419RarmF | ACCGC*ACTAGT*CTCGAGTAGCTATTGCGTCCGTGAATGTC |
| 1419RarmR | GGCGC*GAGCTC*CACCAATCCTCTCTGCTATGATAATC |
| 0558LarmF | ATAGGGCGAATTGGGCCCGACGTCGCATGCAATGTGCCTGTTCTGGTCGATG |
| 0558LarmR | TGGACCATGGCTAATTCCCATATCTACCTCCTCCATTCAATTAAGACAC |
| 0558RarmF | CTTCGAAGCAGCTCCAGCCTCGTCCGACAATACTCATATAGCTTATATCC |
| 0558RarmR | TAGAATACTCAAGCTATGCATCCAACGCGTCAACTGATCGGCAATCTCATCA |
| 1170LarmF | ATAGGGCGAATTGGGCCCGACGTCGCATGCATGGCACGCGAAGCTGATTTA |
| 1170LarmR | TGGACCATGGCTAATTCCCATAGGCATTCCCGTTTTGTATCGTT |
| 1170RarmF | CTTCGAAGCAGCTCCAGCCTCAAATCCAAAAACCGGATAGCC |
| 1170RarmR | TAGAATACTCAAGCTATGCATCCAACGCGTTAGCCTTGATCGACCATATCGG |
| 1414LarmF | ATAGGGCGAATTGGGCCCGACGTCGCATGCGAAGCAAAGATTCTTGCCGATACA |
| 1414LarmR | TGGACCATGGCTAATTCCCATAAGAGCCTACCTTTCTTATTACTTCAGC |
| 1414RarmF | CTTCGAAGCAGCTCCAGCCTAAGATAAAAGCCGGTCTGAAATCC |
| 1414RarmR | TAGAATACTCAAGCTATGCATCCAACGCGTCCGAATAAAGTCCTGACGGTTG |
| 1624LarmF | ATAGGGCGAATTGGGCCCGACGTCGCATGCTTATTCTCGAAGCCCGTCCCA |
| 1624LarmR | TGGACCATGGCTAATTCCCATATTCCGATGGTCAGAAGGGTTGT |
| 1624RarmF | CTTCGAAGCAGCTCCAGCCTTTTGACCGCTTATCTCATTGGC |
| 1624RarmR | TAGAATACTCAAGCTATGCATCCAACGCGTCAGAAAGAGAATGAGCAGTAGCGAT |
| cysIJLarmF | TCACTATAGGGCGAATTGGGCCCGACGTCCGCCAAACAGCGACCTTC |
| cysIJLarmR | GATATTCATATGGACCATGGCTAATTCCCATTATACAAGGACTTTCAGTATCAGCGA |
| cysIJRarmF | AGTATAGGAACTTCGAAGCAGCTCCAGCCTCTGCCGAATTTCTTGACCAA |
| cysIJRarmR | TAGAATACTCAAGCTATGCATCCAACGCGTGGTTCATGGGTTCGACTTTG |
| cysNDLarmF | TCACTATAGGGCGAATTGGGCCCGACGTCGCTTGCGGAACAACTGGAA |
| cysNDLarmR | GATATTCATATGGACCATGGCTAATTCCCATCACTAATCCTTTAAGCCAGATTGAT |
| cysNDRarmF | AGTATAGGAACTTCGAAGCAGCTCCAGCCTTACGATATCAAGATTGCAGGCA |
| cysNDRarmR | TAGAATACTCAAGCTATGCATCCAACGCGTAAATAGCCAAGGGCGTATCG |
| Cm-cysIJND-F | ATGGGAATTAGCCATGGTCCATATGAATATC |
| Cm- cysIJND-R | AGGCTGGAGCTGCTTCGAAGTTCCTATACT |

(Continued)

| CM-0558-F | GTGTCTTAATTGAATGGAGGAGGTAGATATGGGAATTAGCCATGGTCCA |
| --- | --- |
| CM-0558-R | GGATATAAGCTATATGAGTATTGTCGGACGAGGCTGGAGCTGCTTCGAAG |
| CM-1170-F | AACGATACAAAACGGGAATGCCTATGGGAATTAGCCATGGTCCA |
| CM-1170-R | GGCTATCCGGTTTTTGGATTTGAGGCTGGAGCTGCTTCGAAG |
| CM-1414-F | GCTGAAGTAATAAGAAAGGTAGGCTCTTATGGGAATTAGCCATGGTCCA |
| CM-1414-R | GGATTTCAGACCGGCTTTTATCTTAGGCTGGAGCTGCTTCGAAG |
| CM-1624-F | ACAACCCTTCTGACCATCGGAATATGGGAATTAGCCATGGTCCA |
| CM-1624-R | GCCAATGAGATAAGCGGTCAAAAGGCTGGAGCTGCTTCGAAG |
| T-CM-F | ATGGGAATTAGCCATGGTCC |
| T-CM-R | AGGCTGGAGCTGCTTCGAAGTTCCTATACT |
| 0558-GF | CCTTTATGTTCGTGAGAGCGGGT |
| 0558-GR | CGTCTATCTGGGCGTGAAGAATG |
| 1170-GF | ATGTCGATCAGCCCCGAAATT |
| 1170-GR | AGCCGTAAAAACACATACCGAGG |
| 1414-GF | GACGGTGGAACCAATATCTTTCG |
| 1414-GR | AAGAAGGCTCTAACAGCGAACCC |
| 1624-GF | GAGGTAACATCGGCGTGAATCTG |
| 1624-GR | CTTCATGATTATCGGATTGGATGG |
| 0270-GF | GGACATCTGTCAGATGCTACGAC |
| 0270-GR | TCCTAATAACGACTGGACACAG |
| 1419-GF | CCTATGGACAACTCCATTTCATG |
| 1419-GR | GACGATTAAGACCGTATTCGTGA |
| 0970-GF | GATGGTGACGGCTACCTTGTT |
| 0970-GR | AATGATGCTGGCTGTCAGCA |
| cysIJ-GF | AAGCCTCCAACAGATGCACC |
| cysIJ-GR | TCGACTGATCGCGCCGTAAT |
| cysN-GF | GCAATCAAAGACCGCCGTC |
| cysN-GR | TGTTCCGGCGCTTCATAGAC |

**Table. S3 Primers of overexpression experiment.**

| Primers | Sequence（5’-3’） |
| --- | --- |
| 0270-OF | GTTAGGAGAATAAACATGCCAGATCCTATATTAAAAACG |
| 0270-OR | TACGTT*CTGCAG*TTAATTCCTGTTGGATGCCA |
| 1419-OF | TAGGAGAATAAACATGACTGCGCCAAAATCTCTTC |
| 1419-OR | TTCAAC*AAGCTT*TTAAGGCTTTTTCTGTCCTAAATC |
| 0970-OF | GTTAGGAGAATAAACATGACTTTATTTTCGGCTTCTTC |
| 0970-OR | TTCAAC*AAGCTT*TCAATGGGCAGAATGGG |
| 0558-OF | GTTAGGAGAATAAACATGCCAGATCCTATATTAAAAACG |
| 0558-OR | TACGTT*CTGCAG*TTAATTCCTGTTGGATGCCA |
| 1414-OF | TAGGAGAATAAACATGACTGCGCCAAAATCTCTTC |
| 1414-OR | TTCAAC*AAGCTT*TTAAGGCTTTTTCTGTCCTAAATC |
| 1170-OF | GTTAGGAGAATAAACATGACTTTATTTTCGGCTTCTTC |
| 1170-OR | TTCAAC*AAGCTT*TCAATGGGCAGAATGGG |
| 1624-OF | TTAATATAGGATCTGGCATGTTTATTCTCCTAACTTATTAAGTAGCTAT |
| 1624-OR | ACTTGCTCTAGATGTCGATGCCGAGTTGG |
| PGAP-OF1 | ATATCG*GAATTC*TGTCGATGCCGAGTTGGA |
| PGAP-0270-OR | TTAATATAGGATCTGGCATGTTTATTCTCCTAACTTATTAAGTAGCTAT |
| PGAP-OF2 | ACTTGC*TCTAGA*TGTCGATGCCGAGTTGG |
| PGAP-1419-OR | GATTTTGGCGCAGTCATGTTTATTCTCCTAACTTATTAAGTAGCT |
| PGAP-0970-OR | GCCGAAAATAAAGTCATGTTTATTCTCCTAACTTATTAAGTAGC |
| PGAP-OF3 | AGGTCACCAGCTCACCGTCT*GAATTC*TGTCGATGCCGAGTTGG |
| 0970-OR2 | CGAAGGTGAGCCAGTGTGAC*CTGCAG*TCAATGGGCAGAATGGG |
| PGAP-OF1 | ATATCG*GAATTC*TGTCGATGCCGAGTTGGA |
| PGAP-0558-OR | TTAATATAGGATCTGGCATGTTTATTCTCCTAACTTATTAAGTAGCTAT |
| PGAP-OF2 | ACTTGC*TCTAGA*TGTCGATGCCGAGTTGG |
| PGAP-1414-OR | GATTTTGGCGCAGTCATGTTTATTCTCCTAACTTATTAAGTAGCT |
| PGAP-1170-OR | GCCGAAAATAAAGTCATGTTTATTCTCCTAACTTATTAAGTAGC |
| PGAP-OF3 | AGGTCACCAGCTCACCGTCT*GAATTC*TGTCGATGCCGAGTTGG |
| PEZ-F | AATTCGTTGAATCCTGCCTC |
| PEZ-R | TCAGTGCCAACATAGTAAGCC |

**Table. S4 Identification primers of quantitative Real-Time PCR.**

| Primers | Sequence（5’-3’） |
| --- | --- |
| ZCP4_0221-F | TTGGTATCAACAGGAACA |
| ZCP4_0221-R | ATCTGCTGACTTCTCAAT |
| ZCP4_0439-F | CTAAAGCCCAAAGAGGAA |
| ZCP4_0439-R | CAGGATGAAGAAGGATTGT |
| ZCP4_0558-F | GCATCTTTGGCTTTATCA |
| ZCP4_0558-R | ATTGACGATCCTGAATTG |
| ZCP4_0600-F | TGTCGGAGAAGATACTGA |
| ZCP4_0600-R | AACAAGGCTATCCATCAC |
| ZCP4_0601-F | CTATTCATCTGCCAGGTT |
| ZCP4_0601-R | TTAAGCCAACATTGACATTA |
| ZCP4_0708-F | TGGCGAATAATGTCATAGA |
| ZCP4_0708-R | TGGTCGTTATCATTCTTCT |
| ZCP4_0805-F | GCAGATTACGGAATAACA |
| ZCP4_0805-R | TTGAACATTACGACGATTA |
| ZCP4_1170-F | TTATGCTCTATCCGACTT |
| ZCP4_1170-R | CGACCAATAAAGAAATCAG |
| ZCP4_1331-F | CCGTCCGATAATGATACC |
| ZCP4_1331-R | GATGGAATGTCTATGGCTAT |
| ZCP4_1414-F | CCGATGTGGATAAATATG |
| ZCP4_1414-R | GCGTAATAATCAAGGATAT |
| ZCP4_1481-F | GCGTGCTACTTGGGATAA |
| ZCP4_1481-R | TGATATGGATGTGGTGATTGT |
| ZCP4_1624-F | CGCTACCTATCAATACAC |
| ZCP4_1624-R | ATTATCAACATCCGTCAG |
| ZCP4_1625-F | AGATATACGGTAATCAAGA |
| ZCP4_1625-R | CAACAGTAACTACATCA |
| ZCP4_1673-F | TTCACGATAGATCAACTT |
| ZCP4_1673-R | GTATCAGGTAACAGCATA |
| ZCP4_1703-F | GCACCAATCTGAACACAT |
| ZCP4_1703-R | CAACCTGATAAGAGATAATAGCA |
| ZCP4_1707-F | TCTCACGACTTCCATAAC |
| ZCP4_1707-R | TTCCAATACGCTTATCATTC |
| ZCP4_0241-F | CATCGCATAGTGAATAGA |
| ZCP4_0241-R | GTCAGTTACAGATTAAGTC |
| ZCP4_0242-F | CGTCCTAATAATCTATCTATC |
| ZCP4_0242-R | TTATCGCTATCCTTATGG |
| ZCP4_0441-F | TTCGGCATCTTCGTCAATA |

(Continued)

| ZCP4_0441-R | CGGCTGTCTGTCTATGTT |
| --- | --- |
| ZCP4_1209-F | CTAACCAATCGCCAGACA |
| ZCP4_1209-R | TTGCCAGTGAATCCAGAC |
| ZCP4_1212-F | TGAATCCATCCGTGTCTT |
| ZCP4_1212-R | TTTGGCTGCGAGATAAAG |
| ZCP4_1213-F | CGCATTACCCAAATTCATTATC |
| ZCP4_1213-R | AATAAGAGATCAACAAGACCAAT |
| 16S rRNA-F | CGTTGTTCGGAATTACTG |
| 16S rRNA-R | ACTTACCTCTTCTATGTTCTA |

**Table. S5 Logarithmic growth phase gene expression measurements by RNA-Seq.**

| Functional group and gene | Description | Log2 Fold Change in RNA-Seq | |
| --- | --- | --- | --- |
| **Bacterial chemotaxis** |  |  | |
| ZCP4_1147 | methyl-accepting protein IV | -1.0423 | |
| ZCP4_1150 | chemotaxis protein CheR | -1.0247 | |
| **Flagellar assembly** | | |  |
| ZCP4_0645 | flagellar biosynthesis protein FliP | -1.2498 | |

**Table. S6 Primers for gene amplification and sequencing**

| Primers | Sequence（5’-3’） |
| --- | --- |
| ZCP4_0270-SF | TCGCCACTATTCTGCAGA |
| ZCP4_0270-SR | AGATAATTTCGTGGCCTTAT |
| ZCP4_1419-SF | GTCAGATTTTCACCAATCCTC |
| ZCP4_1419-SR | TGTTCTATCGGTCGGGAA |
| ZCP4_1431-SF | TCTATTTTAATTGTTATTGGCA |
| ZCP4_1431-SR | CACACAGTCACAAGCTATC |
| ZCP4_1578-SF | TGAATGACTGCTTCGGTGG |
| ZCP4_1578-SR | CTATAGCCACCAAGCTATGACAC |
| ZCP4_1616-SF | CCCGTTCTCTGCTAT TCA |
| ZCP4_1616-SR | TGCCTATCTTTAAGCCATG |
| ZCP4_0244-SF | AATCAGCAGCGTCAATCC |
| ZCP4_0244-SR | ATGAAGCGGTGAATGAAGC |
| ZCP4_0525-SF | CCAAATTACAGTCGTCATCAA |
| ZCP4_0525-SR | TCTTTTCGTTAATGCACCGT |
| ZCP4_0970-SF | TTGGTATGCGAGTTAAACGG |
| ZCP4_0970-SR | GTTGACGATTTTCTTTGTGC |

**Ref:**

Agrawal M., Wang Y., Chen RR. (2012). Engineering efficient xylose metabolism into an acetic acid-tolerant *Zymomonas mobilis* strain by introducing adaptation-induced mutations. *Biotechnol Lett* 34(10): 1825–1832.

Datsenko KA., Wanner BL. (2000). One-step inactivation of chromosomal genes in *Escherichia coli* K-12 using PCR products. *Proc Natl Acad Sci U S A* 97(12): 6640–6645.
